# Fungal Pancolitis in an Immunocompetent Patient: A Case Report

**DOI:** 10.34172/mejdd.2025.447

**Published:** 2025-10-31

**Authors:** Coana Sukmagautama, Kenneth Tan, Christopher William Purnomo, Tivano Antoni, Alson Bryant Timotius Sinaga

**Affiliations:** ^1^Department of Internal Medicine, Faculty of Medicine, Universitas Sebelas Maret, Surakarta, Indonesia; ^2^North Buton Regional General Hospital, Ereke, Indonesia; ^3^Medical Clerkship, Universitas Sebelas Maret, Surakarta, Indonesia

**Keywords:** Fungal, Colitis, Immunocompetent, Case report, *Candida*

## Abstract

Normal gastrointestinal flora plays a crucial role in protecting against opportunistic pathogens, including bacteria and fungi. Fungal infection, in particular, may occur in patients whose commensal homeostasis is disrupted. Fungal colitis is a rare manifestation of fungal infection of the gastrointestinal tract, particularly in immunocompetent patients. This presents with a mortality rate of 50%, with *Candida* sp. being the most common causative agent. Evidence in this condition is lacking, and case reports can add to the collective information for generalizable information on this condition. This paper presents a rare case of a 63-year-old immunocompetent woman with fungal pancolitis, its presentation, examination, diagnosis, treatment, and response. Also discussed are the difficulties in diagnosing this rare condition, namely, its similar presentation to functional disorders.

## Introduction

 The gastrointestinal tract typically has a certain flora profile, such as bacteria and fungi.^1^ Maintaining the commensal's homeostasis is crucial for providing essential nutrients and protecting against opportunistic pathogens.^2^ When this homeostasis dissolves, it causes an opportunistic infection in the gastrointestinal tract, such as in the stomach or intestine. Fungal infection is an example of a manifestation due to this disruption of homeostasis. The incidence of these pathogens is increasing due to the sustained use of immunosuppressive drugs, chemotherapy, or in immunocompromised patients.^3^

 Fungal colitis is a rare manifestation of a fungal infection in the gastrointestinal tract. It is a threatening disease that is more likely to happen if host immunity is compromised. *Candida sp.* is the most common etiology, with a 50% mortality rate among all cases of fungal colitis.^4,5^ A complete examination and comprehensive treatment of fungal colitis will reduce mortality. However, studies on fungal colitis are not very robust since the cases are few. Case reporting is a way of strengthening the scientific database that can be used to form more generalizable information on this condition. In this case report, we present a rare case of a 63-year-old immunocompetent woman with fungal pancolitis, its presentation, examination, diagnosis, treatment, and response.

## Case Report

 A 63-year-old woman came to the emergency room with a chief complaint of diarrhea 2 weeks before admission. The patient experienced diarrhea six times a day with approximately 200-300 mL for each defecation. The stool had a type 6 Bristol stool chart consistency. The patient denied any history of bloody stool. The patient also complained of sustained dyspepsia a week before admission, which worsened in the last 2 days. The patient has a history of chronic betamethasone use for musculoskeletal pain.

 On arrival at the emergency room (ER), the patient's vital signs were stable as follows: blood pressure 115/70 mm Hg, pulse 90 bpm, respiration rate 20 times per minute, SpO2 97%. General examination revealed an increase in bowel motility (31 times per minute) and tenderness in the umbilical region and both sides of the iliac region. Peripheral edema in both lower extremities was also found. The laboratory investigations revealed leukocytosis (27.350/µL) along with monocytosis (8.1%), neutrophilia (86.2%), and lymphopenia (5%). Elevation of blood urea nitrogen (BUN) (61 mg/dL) and creatinine (1.14 mg/dL) was also present. The electrolyte profile also revealed hypokalemia, hyponatremia, hypochloremia, and hypocalcemia. The patient was later suspected of having inflammatory bowel disease or infection of the intestines. The patient received a 2-g injection of ceftriaxone once daily, a 500-mg injection of metronidazole three times daily, a 40-mg injection of omeprazole twice daily, 100 mg of rebamipide three times daily, and sucralfate three times daily.

 On the third day, a colonoscopy was performed to determine the organic problem. Hyperemic mucosa, along with white plaques, was found starting from the rectum up to the ascending colon. Pathological examination of the biopsied samples of the ascending colon and sigmoid revealed active chronic colitis. KOH staining was also performed on samples of the sigmoid and ascending colon, where spores resembling *Candida *sp. were found. Until the 7th day, the patient was treated with a 500 mg injection of levofloxacin three times daily and a 400 mg injection of fluconazole once daily, due to the finding of a fungal infection in the colon, and 500 mg of sulfasalazine three times daily, which acts as an immunomodulator. After 7 days of hospitalization, the patient was later discharged due to improvement in his condition. Fluconazole, rebamipide, sucralfate, and sulfasalazine were prescribed as take-home medications. The patient was also scheduled for a follow-up re-colonoscopy to evaluate the disease response.

**Figure 1:**
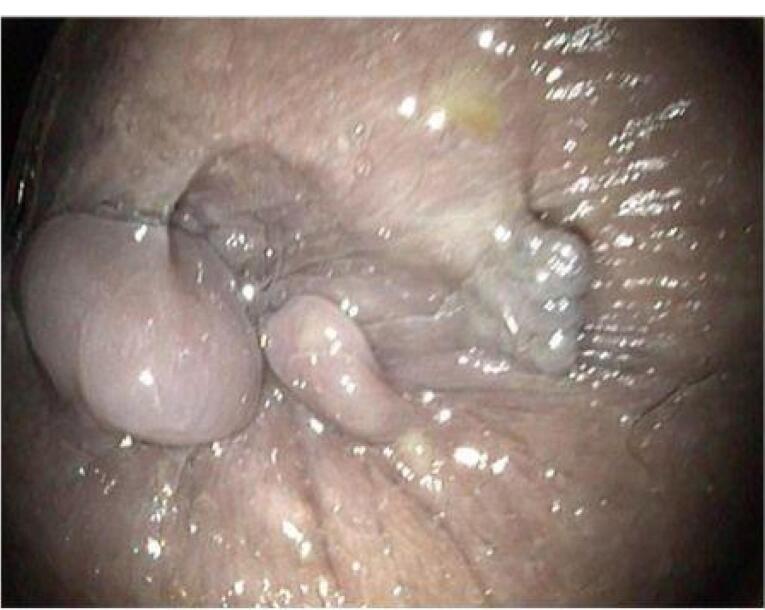
Grade 1 external and internal hemorrhoid, fistula (-), normal anal sphincter tone impression

**Figure 2:**
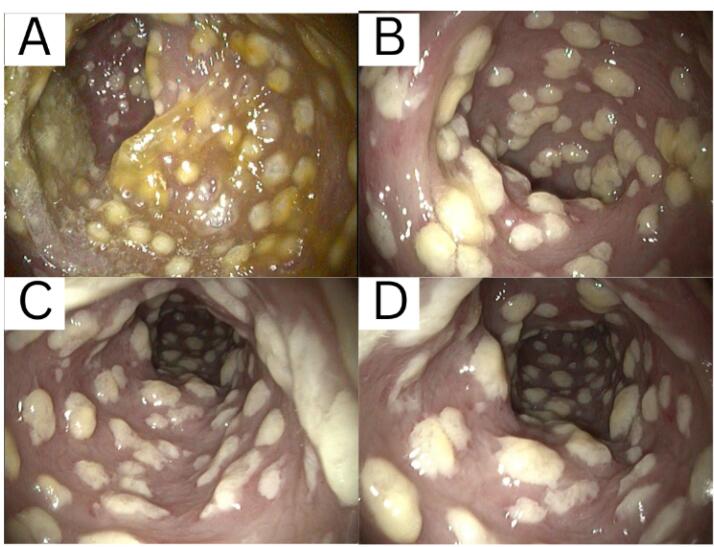
Rectum (A), sigmoid colon (B), descending colon (C-D) with hyperemic mucosa ( + ), edema ( + ), covered with white plaque, plaque tightly attached to mucosa ( + ), erosive ( + )

**Figure 3:**
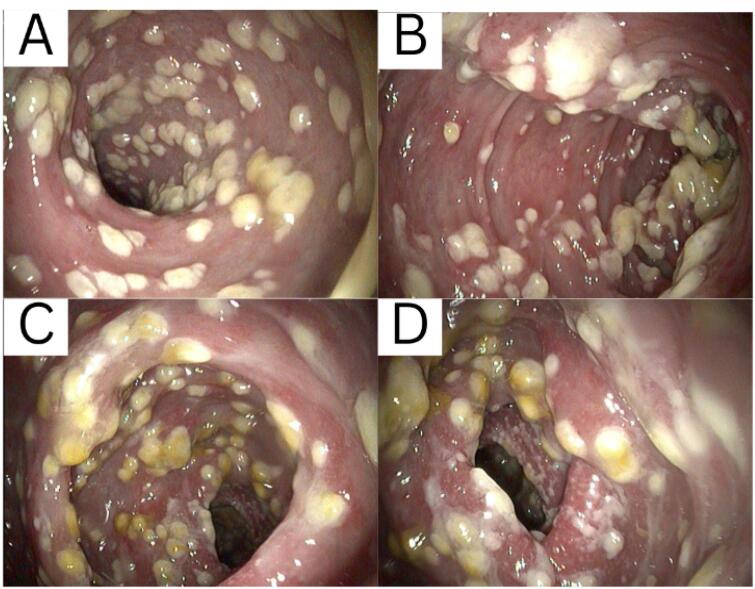
Transverse colon (A-B), ascending colon (C-D) with hyperemic mucosa ( + ), edema ( + ), covered with white plaque, plaque tightly attached to the mucosa ( + ), erosive ( + )

**Figure 4:**
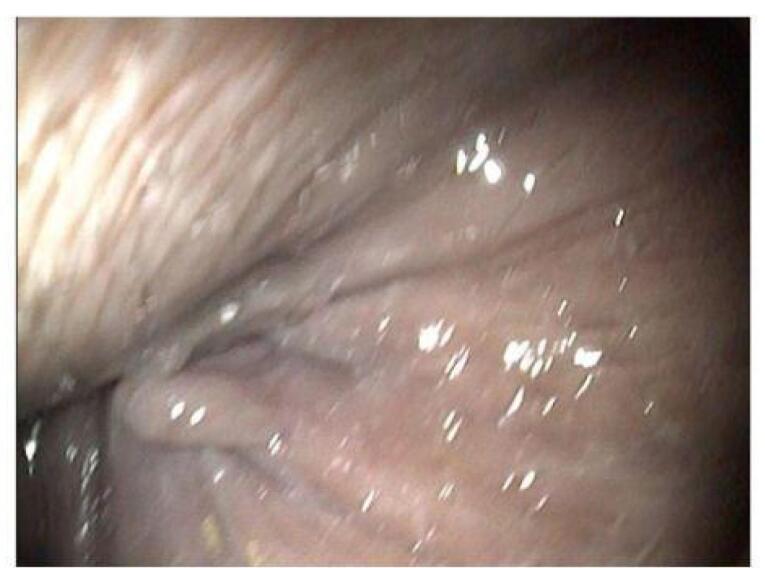
External hemorrhoid view, fistula (-), normal anal sphincter tone impression

**Figure 5:**
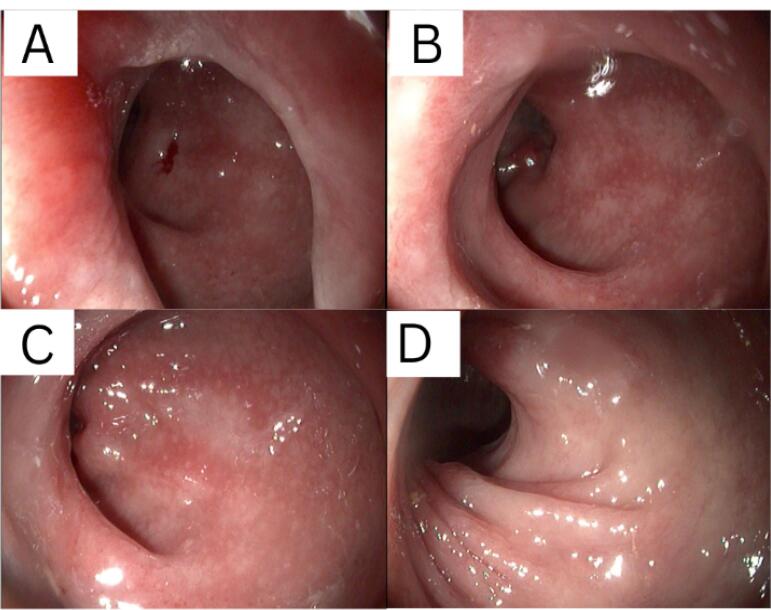
Rectum (A), sigmoid colon (B), descending colon (C-D) with hyperemic mucosa ( + ), erosive ( + ) Mayo criteria 1, ulcer (-), white plaque attached to mucosa (-)

**Figure 6:**
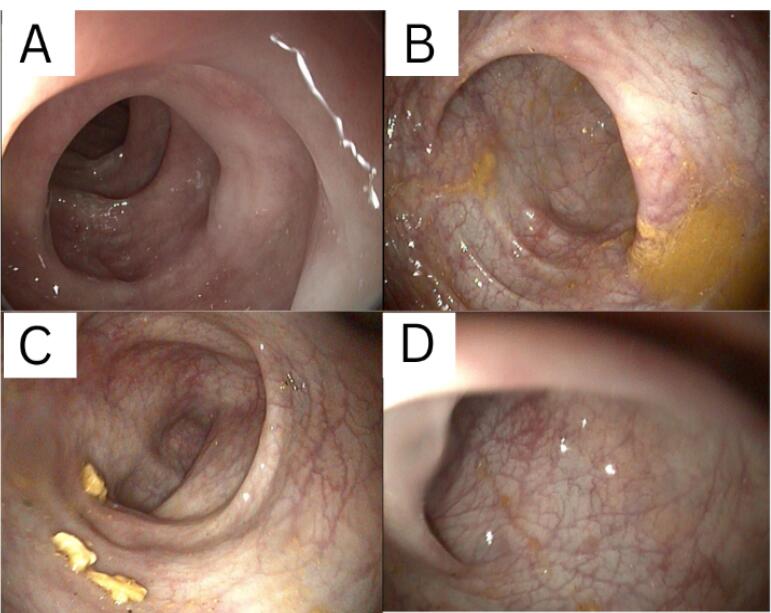
Transverse colon, ascending colon with hyperemic mucosa (-), erosive ( + ) Mayo criteria 1, ulcer (-), white plaque attached to mucosa (-)

**Figure 7:**
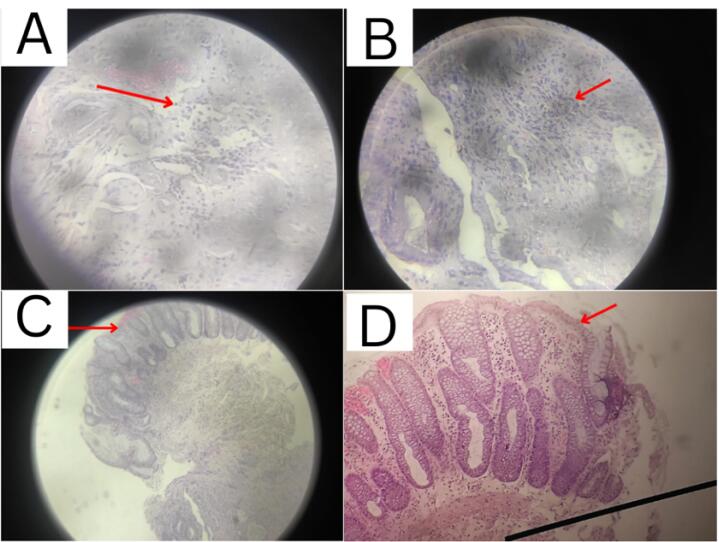
Pathology results showed colonic mucosal tissue fragments with mucosal muscularis, surface epithelium, and partial glands with erosion (C: H&E 4 × and D: H&E 10 × ), and distortion of glands; no atrophy, hyperplasia, or dysplasia were found. Moist stroma, with extravasation of erythrocytes, moderately dissected with lymphocytes and neutrophils (A and B, H&E 40 × )

 From the results of colonoscopy (Figures 1-4), it can be observed that there were grade 1 internal and external hemorrhoids and suspected pancolitis due to fungus. Re-colonoscopy evaluation was performed 4 months later to assess the therapeutic effect of treatment. The mucosa appeared hyperemic and erosive, with Mayo criteria 1 in the rectum area up to the descending colon. The transverse colon to the ascending colon no longer has hyperemic mucosa. The white plaque was no longer found along the colon mucosa (Figure 5). The results of the re-colonoscopy evaluation showed improvement from the first condition, assessed by the significantly reduced fungal colonies (Figures 5 and 6). Pathology examinations were also attached, confirming inflammation, without the presence of neoplasm (Figure 7).

## Discussion

###  Risk Factors for Opportunistic Gastrointestinal Infection

 Infectious colitis, especially fungal, typically presents itself in immunocompromised patients as opportunistic infections.^6^ Whilst reports of fungal colitis in immunocompetent patients are available, this proves to be the exception rather than the rule.^7^ Immunocompromise can be induced by several factors, including chronic diseases, advanced age, and medical treatments. The latter two factors were present in this patient, namely old age (63 years old) and medical treatment (chronic betamethasone use).

 The effect of elder age is well studied, with dramatic changes occurring from the 6th decade of life, followed by progression to immunosenescence. Infections are one of the cardinal signs of immune system aging.^8^ While this most commonly manifests as respiratory infections, gastrointestinal infections remain prevalent, especially in the geriatric population.^9^ It is likely that the patient’s age-related immunosenescence played a significant role in the development of her fungal colitis.

 The effects of long-term steroid therapy, such as betamethasone, on the immune system are well documented and understood.^10^ It is an adrenocortical analog that is an agonist of the corticosteroid hormone receptor. In addition to that, it is long-acting and therefore induces a prolonged state of mild immunosuppression, making the patient more susceptible to opportunistic infections.

###  Clinical Reasoning and Diagnosis

 The differential diagnosis of chronic diarrhea is quite varied, including infections (bacterial, fungal, and viral), functional disorders (such as irritable bowel syndrome), and non-infectious inflammatory conditions (such as inflammatory bowel diseases, including Crohn's disease and ulcerative colitis). The patient had no history of fever, and the laboratory investigations showed leukocytosis, which ruled out functional disorders. This was further confirmed by the presence of red flags such as old age, which further necessitates investigation of organic disorders.^11^ Colonoscopy was performed, and subsequently, cultures and pathological examination of samples.

###  Treatment

 The most common antifungal regimen used for fungal colitis is amphotericin B, which is preferred for most fungal colitis, namely histoplasmosis, penicilliosis, paracoccidioidomycosis, cryptococcosis, and scedosporiosis. However, *Candida *sp. is the most common etiology of large bowel fungal infection. In that case, fluconazole or caspofungin are recommended.^3^ In this report, we administered fluconazole as the antifungal regimen in our patient. There is limited evidence about the efficacy of fluconazole in *Candida* infection in the colon. A case reported by Praneenararat using fluconazole showed clinical improvement, despite the patient’s death due to unrelated comorbidities.^12^

## Conclusion

 Fungal colitis is a rare manifestation of fungal infection in the gastrointestinal tract. The clinical manifestation is similar to other chronic colon infections; therefore, special attention is required for diagnosis and treatment. In our case, we concluded that *Candida* infection in the colon could present with diarrhoea and mimic inflammatory bowel disease. Fluconazole is an effective treatment.
